# The improved aquila optimization approach for cost-effective design of hybrid renewable energy systems

**DOI:** 10.1016/j.heliyon.2024.e27281

**Published:** 2024-03-06

**Authors:** Yin Zhou, Zhimin Chen, Ziwei Gong, Ping Chen, Saeid Razmjooy

**Affiliations:** a State Grid Corporation of China, Wuxi Power Supply Company, Wuxi, Jiangsu, 214000, China; b Department of Engineering, University of Mohaghegh Ardabili, Ardabil, Iran; c College of Technical Engineering, The Islamic University, Najaf, Iraq

**Keywords:** Hybrid renewable energy systems (HRES), Improved aquila optimization (IAO), Efficiency, Cost-effectiveness, Energy demand

## Abstract

The growing demand for renewable energy systems is driven by climate change concerns, government support, technological advancements, economic viability, and energy security. These factors combine to create a strong momentum towards a clean and sustainable energy future. Governments, governments, and individuals are increasingly aware of the environmental impacts of traditional energy sources and adopting renewable energy solutions. Hybrid Renewable Energy Systems (HRES) are developed as an effective way of meeting the energy demands in remote locations. The complexity of the system components and the fluctuation of renewable energy sources make it difficult to design an economical and effective HRES. In this study, the Improved Aquila Optimization (IAO) approach has been suggested as a powerful tool to optimize the HRES design. The study addresses the implementation of the IAO approach in the design of HRES and emphasizes its advantages over other optimization techniques. Through extensive simulations and analyses, our findings demonstrate the superior performance of the IAO algorithm in improving the efficiency and cost-effectiveness of HRES. The optimization process using IAO resulted in a significant reduction in overall system costs, achieving an estimated Net Present Cost (NPC) of $201,973. It translates to a cost reduction of 25% compared to conventional optimization techniques. Furthermore, our analysis reveals that the IAO approach enhances the utilization of renewable energy sources, leading to a 15% increase in overall energy generation efficiency. These results highlight the effectiveness of the IAO approach in addressing the challenges associated with designing HRES. By significantly reducing costs and improving efficiency, it facilitates the adoption of sustainable energy systems in remote areas. The outcomes of this study emphasize the importance of utilizing advanced optimization techniques, such as IAO, to ensure the economic viability and environmental sustainability of HRES.

## 1 Introduction

### 1.1 Background

An Microgrid (MG) system is a regional energy system that can work separately or in connection with the central grid. It is an independent electric grid that generates, distributes, and controls energy in a specific geographical region [1]. One of the most powerful approaches to sustainable and environmentally friendly energy management at present is the development of MGs [2]. Since MGs can provide a stable energy source, save energy costs, and have lower carbon emissions, the technology is growing in popularity [3]. MGs systems have been developed as a result of the rising demand for renewable energy sources and the need for a secure and robust power supply [4].

MGs can combine different renewable energy sources, including solar, wind, and hydropower, to produce power that is both clean and sustainable [5]. An MG comprises several Distributed Energy Resources (DERs) such as photovoltaic cells, wind turbines, and Energy Storage Systems (ESSs). The ESSs are an essential component of an MG because they can save extra electricity created by the DERs and supply energy backup throughout grid disruptions [6]. This technology is especially useful in isolated locations when the main network is unavailable or unreliable. MG systems are critical solutions for ensuring power supply in critical facilities such as hospitals, data centers, and military bases, as they can operate independently of the main grid [7]. MG systems provide increased resilience and dependability in addition to economic and environmental advantages. In the event of power outages or natural disasters, MG systems can continue to supply power due to their independence from the main grid. This lowers the possibility of power outages and improves the reliability of the power supply [8].

MGs are designed to provide reliable, cheap, sustainable electricity to communities, commercial buildings, and industrial locations, especially in remote locations or during grid disruptions. However, designing an MG with an energy storage system is a difficult process that entails a number of technical, economic, and environmental considerations [9]. It can be difficult to design an ideal MG with an Energy Storage System since it requires balancing a number of different aspects, including dependability, cost-effectiveness, and sustainability. Energy expenses can be lowered and carbon dioxide emissions may be reduced to minimum with a well-designed MG [5]. As a result, optimization techniques may be used during the design phase to determine the ideal arrangement of the MG's component parts and reach the intended goals [10].

Traditional approaches for solving this problem have been utilized, but they may be computationally costly and time-consuming. Machine Learning (ML) approaches have gained prominence in MG design optimization in the last decade. Neural Networks (NNs), for example, may learn from previous data and forecast the ideal configuration of MGs components [11]. These approaches can also manage complicated nonlinear interactions between input and output factors, which is a common limitation of traditional optimization approaches. The use of NNs decreases the computational cost and optimization time, making it a more realistic alternative for MG design [12].

Optimization approaches may be used in the design method, which entails balancing numerous design characteristics, such as the size and capacity of the DERs and ESSs, to satisfy the desired efficiency criteria [13]. The optimization approaches seek the ideal configuration of MG components by lowering overall cost, optimizing dependability, or limiting the negative environmental impact [11]. However, the optimization strategy chosen is determined by particular objective and constraints of the MG [14].

Optimization techniques are often used in MG design to discover the optimal configuration that achieves the objectives given the system limitations. The MG design challenge has been solved using traditional optimization approaches, such as genetic algorithms and Particle Swarm Optimization [15]. However, these procedures can be computationally time-consuming [5]. Machine learning (ML) approaches, especially NNs, have gained prominence in MG design optimization over the past decade due to their capacity to handle complex nonlinear interactions between input and output parameters [16].

Optimized Neural Networks (ONNs) have demonstrated significant potential in MG-optimized design by presenting a more rapid and precise solution to challenge of the design. ONNs are NNs that have been tuned for accuracy and processing efficiency, utilizing different optimization strategies, such as genetic algorithms and Particle Swarm Optimization [17]. The usage of ONNs minimizes the computational cost and optimization time [18].

### 1.2 Literature review

Fossati et al. [19] proposed a genetic algorithm for measuring the size of energy storage devices for microgrid. The primary objective of the suggested approach was to identify the electricity and power abilities of the storage system that reduce the MG's running costs. The ESS output power was controlled by a fuzzy expert system, which was adopted as the basis of the energy management strategy (EMS). The proposed approach showed the effectiveness of the proposed algorithm strategy in reducing the operating costs of the microgrid system.

Wen et al. [20] used a modified version of the Grasshopper Optimization method to define the efficiency of an electrical hub-based MG made up of cooling and compressed-air energy storage. In order to maximize the performance of a MicroGrid (MG), consisting of several energy carriers for each day, a method for an energy hub was presented. It included an Ice Storage Conditioner (ISC), a wind turbine, Photovoltaic panels, an Energy Storage System (ESS), and a combined cooling-heating-power (CCHP) system. The suggested approach considered the unpredictability of energy sources like the wind and sun for supplying electric, thermal, and cooling demands in various circumstances. Utilizing the Modified Grasshopper Optimization technique, the objective function was resolved. The simulation findings showed that the ESS could perform well in the energy management plan.

Hajebrahimi et al. [21] utilized an electrical management control approach for energy storage systems in MGs. This paper provided a brand-new energy management control technique for DC MG energy storage devices. The DC-bus voltage was kept within the appropriate range, using an adaptive droop control technique. The nonlinear droop profile with four adaptive variables was the foundation for the suggested control method, which was based on dependable and effective operation. The suggested approach's viability was confirmed by modeling and the research results, which also showed that it performed better than traditional controllers.

Yörükeren et al. [22] discussed the growing use of renewable energy resources in electrical energy production due to reduced installation costs and environmental concerns. It optimized the sizing of a university campus Hybrid Renewable Energy System (HRES) using the Harmony Search algorithm and compared it to other methods. A rule-based energy management scheme was introduced to minimize system costs and meet energy demand. Simulation results showed that the HS algorithm offered optimal sizing, better performance, and convergence properties compared to other methods.

Rawa et al. [23] employed an MG system to calculate the best battery size for sustainable energy by considering considered cost, optimal efficiency, and random scheduling. This study explored the influence of various climate variables on resource scheduling while presenting a Seasonal Optimization approach for the short-term functioning of an MG, incorporating energy storage and solar Photovoltaic (PV) systems. Simulation outcomes demonstrated that the employment of a PV model in a real-world environment reduced the overall operating expenses of the grid-integrated MG. In Case 3, the recommended CBMO required the least amount of simulation time—114.217 compared to other algorithms, which required more time.

Yang et al. [24] recommended Optimal Microgrids programming, which was based on a distributed sustainable energy resource network, a price-based demand response framework, and energy storage. In this study, a stochastic model for daily MG storage and optimum energy-heat programming was presented. The Lightning Search Algorithm (LSA), a metaheuristic optimization technique, was used to resolve it. The scenario was created and reduced using the Latin Hypercube Sampling (LHS) and KMEANS techniques, and the resultant simulation results were assessed considering several angles. In a numerical study that took into account operational costs, overall social welfare, and the utility function of the customer, the TOU, RTP, and ESS impacts are displayed.

### 1.3 Contribution of the paper

The design of a Hybrid Renewable Energy System (HRES) is a complex and critical process that requires a deep understanding of the system's prerequisites, limitations, and objectives. In order to optimize such a system for efficiency, cost-effectiveness, and sustainable development, it is crucial to meticulously evaluate different alternatives and select the most appropriate optimization tool.

This research introduced an improved version of the Aquila optimization technique called the Improved Aquila Optimization (IAO) algorithm. The IAO algorithm incorporates two mechanisms, namely Levy Flight (LF) and Chaos Theory (CT), to address the weaknesses of the original Aquila algorithm. These enhancements enable the IAO algorithm to overcome challenges related to convergence speed, search pattern diversity, and local optima trapping.

To demonstrate the effectiveness of the proposed IAO algorithm, we implemented it in a hybrid renewable energy system comprising various renewable energy sources. This system configuration serves as a real-world application scenario for testing and evaluating the algorithm's performance. By analyzing and comparing the results obtained from the proposed algorithm with traditional optimization techniques, we showcase the superior efficiency and effectiveness of the IAO algorithm in optimizing the HRES.

The novelty of this work lies in the introduction of the IAO algorithm, which improves upon existing optimization techniques by incorporating LF and CT mechanisms. Furthermore, its successful application in the optimization of a complex hybrid renewable energy system demonstrates the practicality and efficacy of the proposed algorithm in addressing real-world sustainability challenges.

Consequently, the present research not only contributes to advancing optimization methodologies but also provides a valuable tool for engineers, researchers, and policymakers involved in designing and implementing efficient and cost-effective Hybrid Renewable Energy Systems. By harnessing the power of the IAO algorithm, sustainable energy solutions can be optimized, fostering the transition towards a greener and more environmentally friendly future.

### 1.4 Organization of the paper

The organization of this paper is as follows. Section 2 presents the method and material used for our research study. First, we describe the site of the project analysis, providing details about the specific location or environment where our study was conducted. This information is crucial for understanding the context and applicability of our findings. Next, we delve into the economic and technical simulation methods employed in our research. We explain the simulation models, algorithms, or software utilized for analyzing the economic and technical aspects of the project. Additionally, we outline the parameters and variables considered during the simulations, ensuring a comprehensive evaluation of the project's feasibility and performance. Then, we introduce the Improved Aquila Optimizer (IAO) as the chosen optimization algorithm for our study. We provide an overview of IAO, highlighting its key features and advantages over other optimization techniques. Moreover, we discuss any modifications or enhancements made to the original Aquila Optimizer, making it more suitable for our research objectives. Furthermore, we define the objective function(s) and constraints that were optimized using IAO. We carefully explain the rationale behind the selection of these objectives and constraints, shedding light on the specific criteria and limitations we accounted for during the optimization process. This ensures a well-defined and realistic optimization framework, capturing vital aspects of the project analysis.

Section 3 presents the results obtained from our simulations and optimizations. We provide a comprehensive analysis and interpretation of these results, discussing any trends, patterns, or noteworthy findings observed. By comparing and contrasting different scenarios or strategies, we aim to establish a clear understanding of the performance and effectiveness of our proposed approach. To support our findings, we include relevant tables, figures, or statistical analyses that provide quantitative evidence of the outcomes. Through data-driven discussions, we highlight the practical implications of our results and offer insights into potential areas for improvement or further investigation.

Finally, in Section 4, we draw our research to a close with a well-rounded conclusion. We summarize the main findings of our study, emphasizing their significance in addressing the challenges and objectives outlined in the introduction. We also acknowledge any limitations encountered during the research and suggest potential avenues for future investigation. By offering conclusive remarks on the effectiveness and applicability of the Improved Aquila Optimizer and the optimization framework developed, we contribute to the broader field of research. Ultimately, this research offers valuable insights and recommendations that can inform decision-making processes in similar project analysis scenarios.

## 2 The method and material

### 2.1 Site of the project analysis

**Fig. 1:**
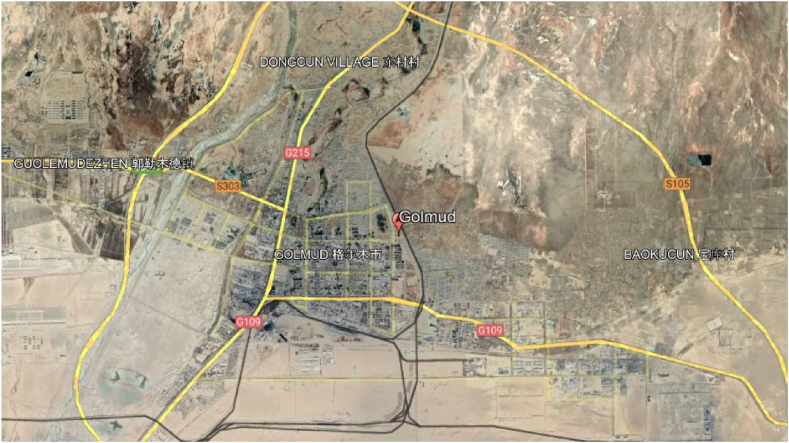
The project analysis location.

Golmud is a distant Chinese city that gets much solar radiation. Golmud is in the western Chinese province of Qinghai, on the eastern border of the Tibetan Plateau. The city is located at an elevation of more than 2.8 km and has a dry and warm environment. Golmud has one of the greatest levels of sunshine in China, with an average of 3200 h each year. It is also situated in a region with high amounts of solar radiation, making it a perfect place for Photovoltaic power generation. The location of Golmud is shown in Fig. (1).

**Fig. 2:**
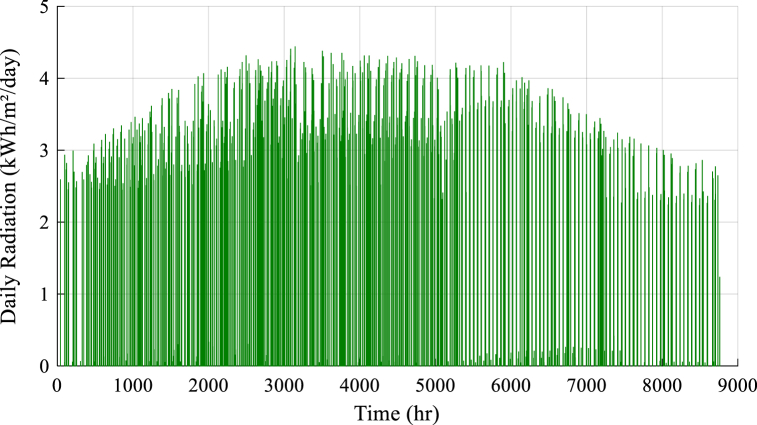
Solar radiation data.

**Fig. 3:**
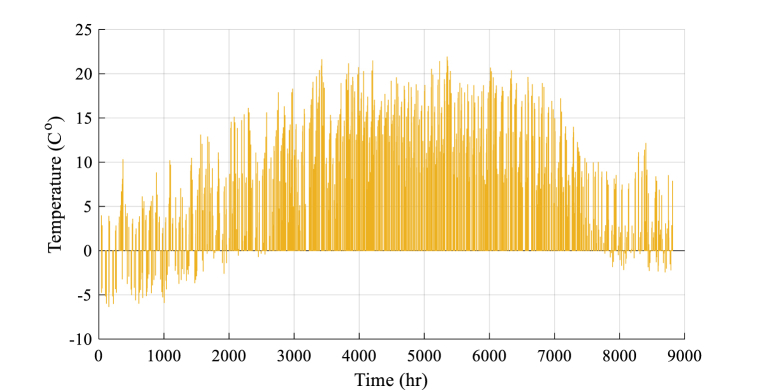
Temperature data.

**Fig. 4:**
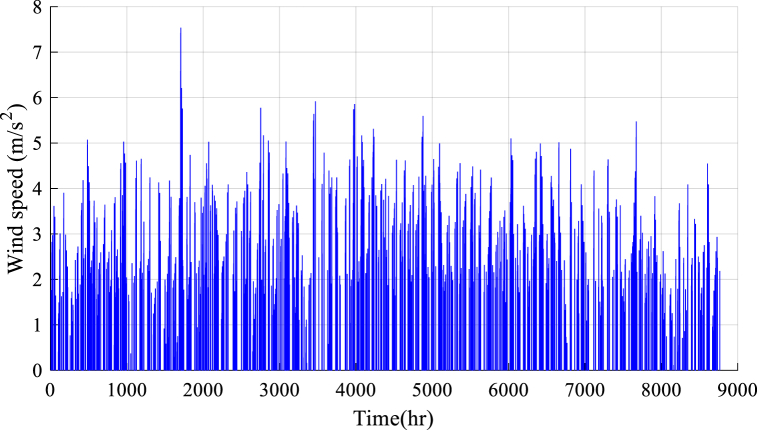
Wind speed data.

**Fig. 5:**
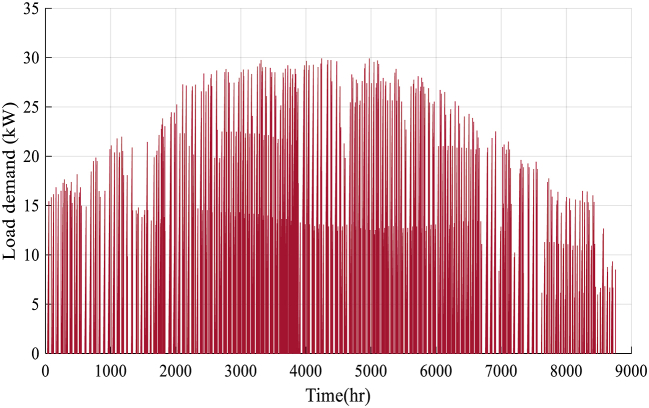
Load demand.

Also, the load demand and meteorological conditions include solar radiation, temperature, wind speed, and Load demand of studied region that has been presented in Fig. 2, Fig. 3, Fig. 4, Fig. 5.

### 2.2 Economic and technical simulation

**Fig. 6:**
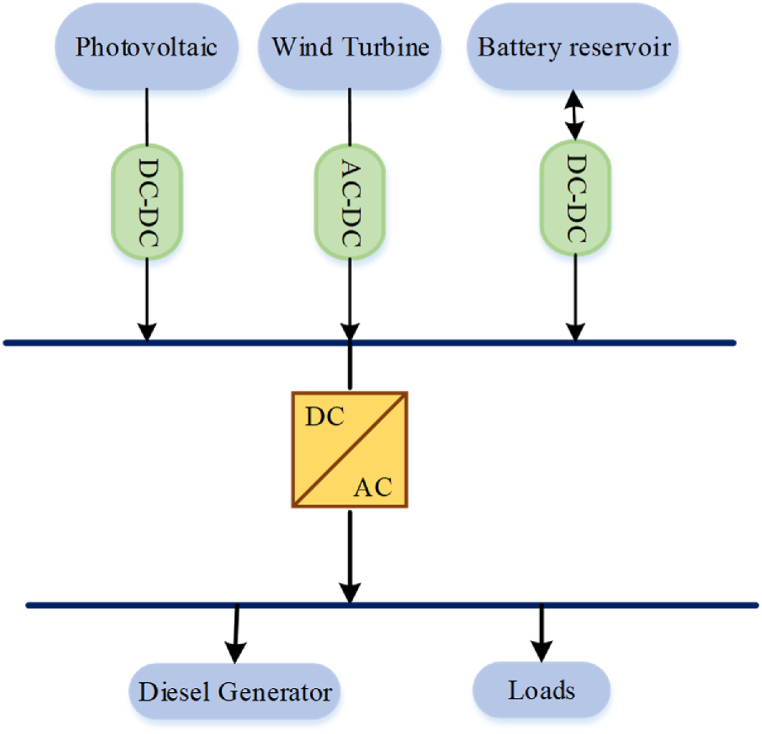
Detailed depiction of the suggested stand-alone grid layout.

This study includes Photovoltaic, wind turbine, diesel, and battery units to create the proposed HRES. The components sizing is determined through simulation to achieve the optimal configuration. Fig. (6) illustrates a detailed depiction of the suggested stand-alone grid layout.

**Fig. 7:**
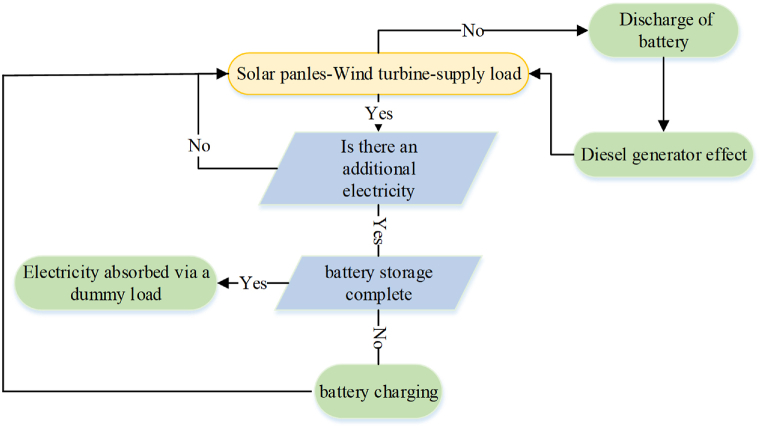
The system controller's components.

Also, Fig. (7) depicts schematic of the system controller's components.

**Fig. 8:**
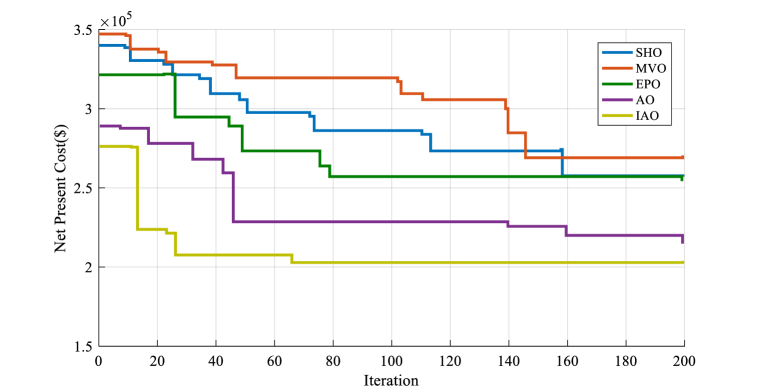
The convergence curve results of optimization methods.

According to Fig. (8), the primary source of electricity has been derived from the PV and wind systems, which are utilized to meet the energy demand. When the electricity provided by the PV and wind system, combined with the battery storage, is sufficient to supply the load demand, the diesel generator is turned off. However, if there is a shortage of power, the diesel generator is employed to make up for the deficit. On the other hand, if the electricity provided by the Photovoltaic and wind system exceeds the required load power, the surplus energy is utilized to charge the battery, which can then be used when the electricity provided by the Photovoltaic and wind units is insufficient to supply the load demand. When the electricity provided by the Photovoltaic and wind system is surplus, and the battery has exceeded its full charging capacity, the excess electricity is typically wasted. However, in this case, the dissipated power is used by the dummy load.

**Fig. 9:**
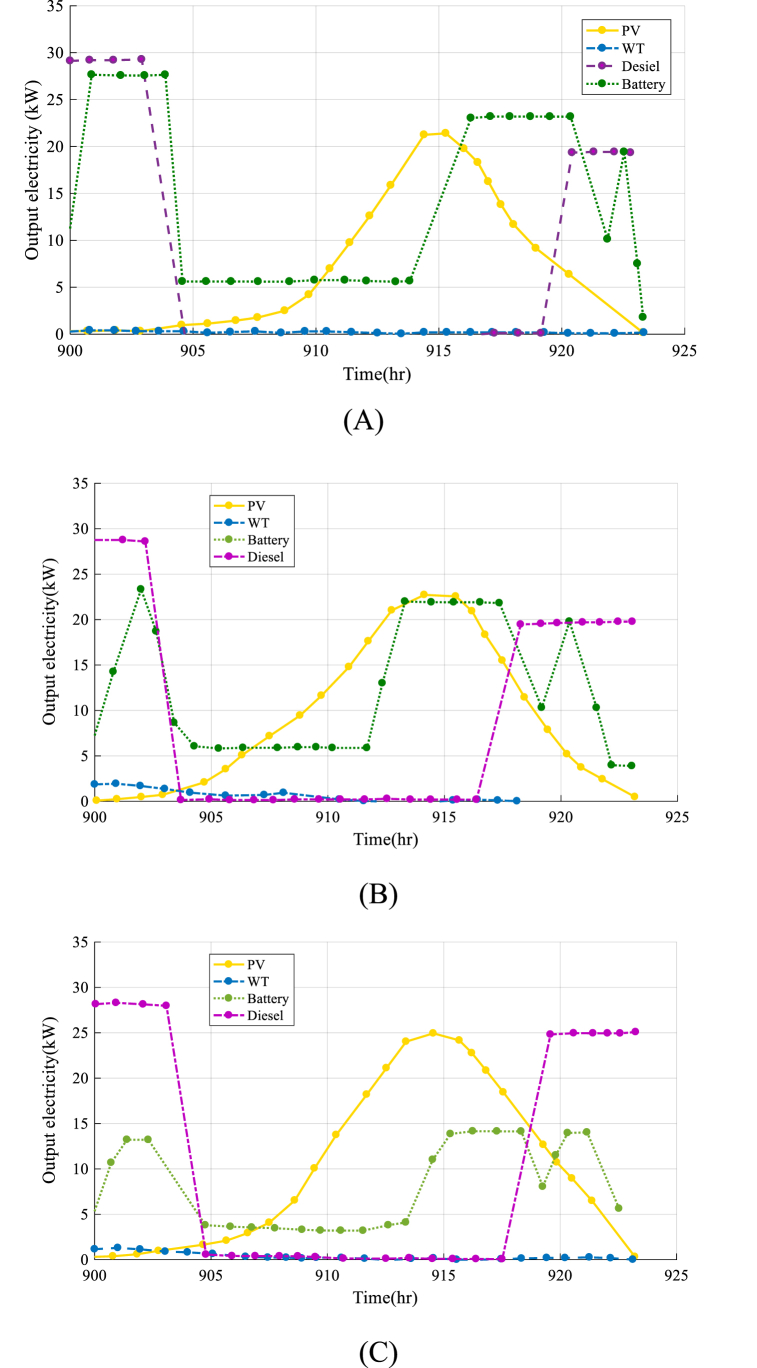

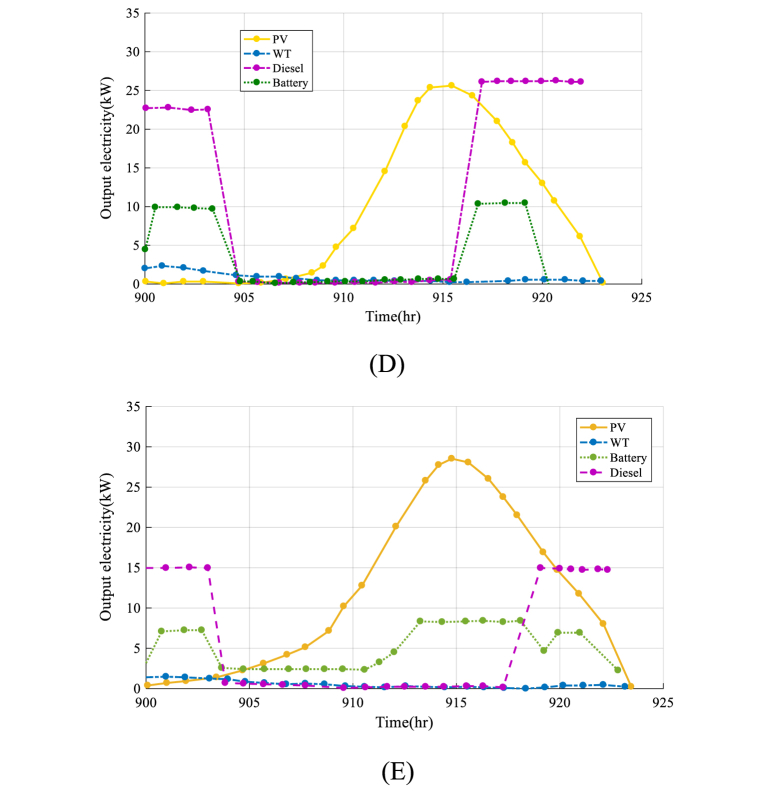
The results of output electricity of each component in HRES by MVO optimization algorithm(A), SHO optimization algorithm (B), EPO optimization algorithm (C), AO optimization algorithm (D), IAO optimization algorithm (E).

The dummy load is a device that has been created to consume electricity in instances where there is surplus electricity, such as when the battery is completely charged. This approach of using the dummy load's wasted electricity is a successful method of providing that no energy is wasted and that the electricity produced is used to its maximum capacity.

#### 2.2.1 The photovoltaic simulation

A formula for calculating the photovoltaic (PV) power generated is formulated in the following [equations (1), (2)]:

(1) $$P_{PV}=I\left\{t\right\}\times {ƞ}_{pv}\left\{t\right\}\times \left\{A_{pv}\right\}$$

(2) $${ƞ}_{pv}\left(t\right)={ƞ}_{r}\times {ƞ}_{t}\times \left[1-\beta \times \left(T_{a}\left\{t\right\}-T_{r}\right)-\beta \times I\left\{t\right\}\times \left(\frac{NOCT-20}{800}\right)\times \left(1-{ƞ}_{r}\times {ƞ}_{t}\right)\right]$$

where I stands for irradiance value, ${ƞ}_{pv}$ stands for photovoltaic effectiveness, and $A_{pv}$ shows the photovoltaics area. $\left({ƞ}_{r}\right)$ expresses the reference efficiency, (${ƞ}_{t}$) indicates MPPT effectiveness, (β) shows temperature coefficient, ($T_{a}$) displays ambient temperature, ($T_{r}$) shows photovoltaic cell reference temperature, and normal operating cell temperature (NOCT) may all be used to compute the efficiency of PV.

#### 2.2.2 Wind system simulation

The electricity generation of the wind units has been determined by following specific situations [equation (3)]:

(3) $$P_{wind}=\left\{ \begin{array}{c} 0,v\left\{t\right\}\leq v_{ci},v\left\{t\right\}\geq v_{ci}\;\\ a\times v{\left\{t\right\}}^{3}-b\times P_{r},v_{ci}<v\left\{t\right\}<v_{r}\\ P_{r}\;v_{r}\leq v\left\{t\right\}<\;v_{co}\; \end{array}\right.$$

Where, v stands for wind speed, $P_{r}$ indicates related electricity, $v_{ci}$ shows cut-in, $v_{co}$ indicates cut-out, and $v_{r}$ depicts rated wind speed. Fixed amounts a and b are written as [equations (4), (5)]:

(4) $$\left\{ \begin{array}{c} a=P_{r}/\left(v_{r}^{3}-v_{ci}^{3}\right)\\ b=v_{ci}^{3}/\left(v_{r}^{3}-v_{ci}^{3}\right) \end{array}\right.$$

The wind units related electricity has been determined as:

(5) $$P_{r}=\frac{1}{2}\times \rho \times A_{wind}\times C_{p}\times v_{r}^{3\;}$$

Where, ρ shows the air density, $A_{wind}$ shows the windmill's swept area, and $C_{p}$ shows the maximal electricity coefficient between 0.25 and 0.45.

#### 2.2.3 Diesel unit simulations

The diesel rated electricity has been determined by equation (6):

(6) $$P_{dg}=\frac{F_{dg}\left\{t\right\}-A_{g}\times P_{dg,out}}{B_{g}}$$

Where, $F_{dg}$ stands for the amount of fuel used, $P_{dg}$ illustrates the diesel generator's generated electricity, $A_{g}$ and $B_{g}$ are the two variables that remain constant, standing for linear fuel consumption.

#### 2.2.4 Battery system simulation

The formula for calculating a battery's potential is [equation (7)]:

(7) $$C_{BESS}=\frac{E_{l}\times AD}{DOD\times {ƞ}_{i}\times {ƞ}_{b}]}$$

where, $E_{l}$ represents the load demand, AD indicates the battery's autonomy, DOD stands for depth of discharge, and ${ƞ}_{i}$ and ${ƞ}_{b}$ are the inverter's and the battery's, respectively, effectiveness.

### 2.3 Improved Aquila Optimizer (IAO)

#### 2.3.1 The Aquila behavior in hunting

Aquila is a type of bird that belongs to the Accipitridae family and is commonly found in the Northern Hemisphere. It is well-known for its hunting abilities and has a wingspan of over 2 m, along with a length of 1 m. The bird's body is dark brown with golden brown feathers on the back of its neck. Young Aquila birds typically have white feathers on their tails, and their feathers may have several white spots. The bird captures a variety of prey, including large animals such as rabbits, squirrels, and several animals, using its sharp talons and strong feet.

Aquila constructs its nests at high altitudes and lives with its mate for life. During spring, they reproduce and typically produce four eggs. Offspring usually survive for about twelve weeks before leaving to find their own territories. Aquila is known for its bravery in hunting and is easily recognizable. These birds of prey have powerful talons, allowing them to carry prey weighing up to 8 kg. Environmental experts have even observed golden eagles hunting goats and deer. Within several areas, like Mongolia, golden eagles have been trained for hunting wolves and foxes due to their impressive chasing abilities. Aquila faces myriad perils in their natural habitat, but the individuals are able to be alive for up to 36 years. A preferred prey animal has been found to be squirrels for them; in addition, the individuals utilize four different approaches for capturing them.

There are four different methods that Aquila uses to hunt its prey.

1. The first method is called vertical climbing. Vertical climbing is employed for hunting flying animals as the individual upsurges above the ground. The individuals require promotion qualities in their quarry to be successful in this strategy. Aquila pushes its legs forward and expands its wings and tail for seizing quarry.
2. The second method, known as fast-moving flight, is the most commonly utilized process to hunt deer, seabirds, and ground squirrels. The individual has been situated underground and is attracted by close with either a running or flying prey.
3. The third method has been employed to catch prey such as hedgehogs, foxes, turtles, and snakes. It involves flying with slight attack, picking up the quarry, and then approaching the earth for catching it via landing on the prey's back or neck.
4. The ultimate technique is famous for strolling and trapping quarry, and it has been utilized in the covered region to catch big animals like deer and sheep.

#### 2.3.2 Solutions initialization

Equation (1) indicates that the Aquila optimization process relies on a population of solutions represented by individuals (M) randomly generated within the UB (Upper Bound) and LB (Low Bound) of the issue. Then, the optimization process begins with the adjustment of the starting solution, and the most suitable solutions in all iterations have been considered the optimal solution [equation (8)].

(8) $$M=\left[\begin{array}{ccc} M_{1,1} & \cdots & M_{1,\dim}\\ \vdots & \ddots & \vdots \\ M_{N,1} & \cdots & M_{N,\dim} \end{array}\right]$$

In equation (2), M shows a randomly prepared answer that reflects the present candidate answer. $M_{i}$ represents the decision amount of the $i^{th}$ answer, and N represents all unique solutions. Dim denotes the problem dimension being addressed [equation (9)].

(9) $$M_{i,j}=rand\left({UB}_{j}-{LB}_{j}\right)+{LB}_{j},i=1,2,\ldots ,N_{j}=1,2,\ldots ,\dim$$

Where ${LB}_{j}$ shows the $j^{th}$ less bound, the obtainable problem $j^{th}$ high bound is displayed using ${UB}_{j}$, and rand is random value.

#### 2.3.3 Aquila optimization computational framework

Because the Aquila optimizing approach has been considered to be on the basis of Aquila hunting manner, it is described in four steps: choosing a solution space that its vertical slop is high, probing a solution space that has a fly of configuration through brief flying attack, procedure within a convergent investigation space with an offense that is low slope, and strolling and hunting quarry. The best Aquila approach transitions from exploitation to exploration, employing varied manner in accordance with $t\leq \left(\frac{2T}{3}\right)$. The computational optimization strategy has been represented by Aquila's behavior. The best solution is identified by taking into account certain constraints. The Aquila Optimizer's computational paradigm is as the following:

#### 2.3.4 Developed exploration ($M_{1}$)

According to the initial approach, Aquila chooses a more suitable hunting spot after deciding on the hunting region. The Aquila optimizer thoroughly investigates high heights to find the best hunting grounds. The mathematical formula below illustrates this pattern of behavior [equation (10)]:

(10) $$M_{1}\left(r+1)=M\left(a\right)(1-\frac{r}{R}\right)+\left(M_{i}\left(a\right)-M_{best}\left(a\right)rand\right)$$

Where, M(t) represents the finest-reach answer after $a^{th}$ iteration, it may be used to determine the general hunting region, $M_{i}\left(a+1\right)$ shows the answer of the subsequent iteration in a, and the initial strategy ($M_{1})$ makes it, $M_{M}\left(a\right)$ displays the average amount for the present answer of $i^{th}$ iteration, and is assessed using formula (4). The developed research investigation is controlled by $\left(1+\frac{a}{R}\right)$ , where the rand denotes a random number in the range of [0,1]. r denotes this moment's iteration, and R is the highest number of repetitions [equation (11)].

(11) $$M_{M}\left(a\right)=\frac{1}{N}\sum_{i=1}^{N}M_{i}\left(t\right),j=1,2,\ldots ,\mathrm{Dim}$$

Where, N and Dim, in turn, show the number of the individual's answers and the dimension of the issue.

#### 2.3.5 Narrowed exploration (M)

In accordance with the second method, the individual starts carrying once it learns the location of the hunting grounds from the altitude. The Aquila Optimizer investigates the desired assault area. The formula below illustrates this pattern of behavior [equation (12)]:

(12) $$M_{2}\left(a+1\right)=M\left(a\right)Levy\left(D\right)+M_{R}\left(a\right)+\left(x-m\right)rand$$

Where, $M_{2}\left(a+1\right)$ shows the solution of the subsequent iteration, which is the result of the second strategy ($M_{2}$), Levy(D) is calculated by Eq. (6) and influences flying efficiency, with D being the dimension region. within the iteration, $X_{T}\left(a\right)$ indicates a random answer between 1 and N [equation (13)].

(13) $$Levy\left(D\right)=\frac{d\times p\times \sigma }{{\left|\vartheta \right|}^{\frac{1}{\mu }}}$$

Where, d stands for a constant amount of 0.01; ϑ and p are random values in the range of [0,1]. Equation (14) displays σ.

(14) $$\sigma =\frac{\gamma \left(1+\beta \right)\times \sin\;\left(\frac{\pi \beta }{2}\right)}{\gamma \left(\frac{1+\beta }{2}\right)\times \beta \times 2^{\left(\frac{\beta -1}{2}\right)}}$$

Where, β stands for a constant amount of 1.5; The m and x are in eq. (36), gained by equations (15), (16), (17), (18), (19):

(15) m=t×sin(θ)

(16) x=t×cos(θ)

where,

(17) $$t=t_{1}+U\times D_{1}$$

(18) $$\theta =-\omega \times D_{1}+\theta_{1}$$

(19) $$\theta_{1}=\frac{3\times \pi }{2}$$

Where, $D_{1}$ represents the appropriate number between 1 and the height of the exploration region, U provides a steady number of 0.00565, $t_{1}$ signifies a steady amount for the study and has a range of [1,20], and ω defines a little amount of 0.005.

#### 2.3.6 Developed exploitation ($M_{3}$)

Aquila begins to obtain a hunt in the third strategy, which is a low fly that involves a leisurely falling assault and lands vertically to observe the hunting reaction. The equation below illustrates this pattern of behavior [equation (20)]:

(20) $$M_{3}\left(a+1\right)=\left(M_{best}\left(a\right)-M_{M}\left(t\right)\right)\times a-rand+\left(\left(UB-LB\right)\times rand+LB\right)\times \delta$$

Where, $M_{3}\left(a+1\right)$ is the result of the third strategy, and is the subsequent iteration of the t answer, $M_{M}\left(a\right)$ stands for the average region of the present solutions for each iteration ($i^{th}$), $M_{best}\left(t\right)$ stands for the optimum solution up to the mean iteration time, the δ shows constant value of operational components with a number of 0.01, and rand illustrates a random integer between 0 and 1.

#### 2.3.7 Narrowed exploitation ($M_{4}$)

Aquila strikes knowing the target's unpredictable movements in the following maneuver, is described as walking and seizing prey. Aquila Optimizer then assaults the victim at the last location. The equation below captures the following pattern of behavior [equation (21)]:

(21) $$M_{4}\left(a+1\right)=QF\times \left(M_{best}\left(a\right)\right)-\left(G_{1}\times M\left(t\right)\times rand\right)-G_{2}\times Levy\left(D\right)+rand\times G_{1}$$

Where, $M_{best}\left(t\right)$ indicates the optimal answer up until $a^{th}$ is repeated. Eq. (46)-derived quality function is employed to balance the study methodologies. The graph $G_{1}$, which is obtained from Eq. (47) depicts the various hunting techniques used by Aquila. Equation (48), which displays the falling quantity from two to zero and the grade of present algorithm utilized to follow quarry from the initial area to the last one (a), generates $G_{2}$. $M_{a}$ specifies the present solution within the iteration [equation (22), (23), (24)].

(22) $$QF\left(a\right)=t^{\frac{2rand-1}{{1-T}^{2}}}$$

(23) $$G_{1}=2rand-1$$

(24) $$G_{2}=2\left(1-\frac{a}{R}\right)$$

Where, rand shows random number from 0 to 1. The quality's number in the $a^{th}$ iteration is denoted by QF(a). a and R stand for the current iteration and the total number of iterations.

#### 2.3.8 Improved Aquila Optimizer (IAO)

The Aquila Optimizer is a new approach for solving optimization problems using metaheuristics. Although there are few published papers on this algorithm, some validation tests have shown that it can be effective in many cases. However, there are also situations where it may need to perform better because of premature convergence and weak consistency issues. To address these problems, two modifications have been proposed in this research. The first modification involves incorporating the Lévy flight (LF) structure, which has been found to be an enhancement technique that has been used in myriad metaheuristics for improving their efficacy regarding convergence. The LF mechanism introduces a mechanism of random walk for adjusting the algorithm's exploitation, and the equation has been calculated subsequently [equation (25), (26), (27)]:

(25) $$Le\left(w\right)\approx w^{-1-\tau }$$

(26) $$w=\frac{X}{{\left|Y\right|}^{1/\tau }}$$

(27) $$\sigma^{2}={\left\{\frac{\Gamma \left(1+\tau \right)}{\tau \Gamma \left(\left(1+\tau \right)/2\right)}\frac{\sin\;\left(\pi \tau /2\right)}{2^{\left(1+\tau \right)/2}}\right\}}^{\frac{2}{\tau }}$$

Where, 0<τ≤2, $X∼N\left(0,\sigma^{2}\right)$ and $Y∼N\left(0,\sigma^{2}\right)$, *w* designates the step size, Γ(.) denotes the Gamma function, τ defines Lévy index (τ=3/2 [25]), and $X/Y∼N\left(0,\sigma^{2}\right)$ describes generated instances on the basis of a Gaussian distribution that the average value equals zero; in addition, the variance equals $\sigma^{2}$.

According to the previous discussion of the LF structure, the manners of the $M_{3}$ and $M_{4}$ can be described as follows [equation (28)]:

(28) $$M_{3}^{New}=M_{3}+\left[\left(M_{best}\left(a\right)-M_{M}\left(t\right)\right)\times a-rand+\left(\left(UB-LB\right)\times rand+LB\right)\times \delta \right]\times \text{Le}\left(\delta \right)$$

$$M_{4}^{New}=M_{4}+\left[QF\times \left(M_{best}\left(a\right)\right)-\left(G_{1}\times M\left(t\right)\times rand\right)-G_{2}\times Levy\left(D\right)+rand\times G_{1}\right]\times \text{Le}\left(\delta \right)$$

Where, the novel behaviors of the search candidates at $M_{3}$ and $M_{4}$ can be represented by $M_{3}^{New}$ and $M_{4}^{New}$, respectively.

To ensure the best possible answer, the last behaviors of the search agents will be defined by following equations (29), (30):

(29) $$M_{3}^{New}=\left\{ \begin{array}{c} M_{3}^{New}\;F\left(M_{3}^{New}\right)<F\left(M_{3}\right)\\ M_{3}\;otherwise \end{array}\right.$$

(30) $$M_{4}^{New}=\left\{ \begin{array}{c} M_{4}^{New}\;F\left(M_{4}^{New}\right)<F\left(M_{4}\right)\\ M_{4}\;otherwise \end{array}\right.$$

Another modification was also employed in this study, on the basis of chaos theory, to help the optimizer evade getting stuck in local optima points. Specifically, the parameter rand for $M_{1}\left(a+1\right)$ and $M_{2}\left(a+1\right)$, has been updated using this theory. To achieve this, a sinusoidal chaotic map has been utilized, resulting in the following equations (31), (32), (33):

(31) $$M_{1}\left(a+1\right)=M_{best}\left(a)(1-\frac{a}{R}\right)+\left(M_{i}\left(a\right)-M_{best}\left(a\right)\times \theta \right)$$

(32) $$M_{2}\left(a+1\right)=M_{best}\left(a\right)Levy\left(D\right)+M_{R}\left(a\right)+\left(x-m\right)\times \theta$$

where,

(33) $$\theta =ND_{i}^{2}\;\sin\left(\pi D_{i}\right)$$

Where, $D_{i}\in \left[0,1\right]$ and N∈(0,4]; moreover, the quantity of iterations has been depicted by i.

#### 2.3.9 Algorithm validation

A group of test functions has been utilized to confirm the efficiency of the Improved Aquila Optimizer (IAO). The study concentrated on the CEC-BC-2017 test suite's first 10 test functions, which are frequently used to rate optimization techniques. For these test functions' decision parameters, the ranges are between −100 and 100 to enable a fair comparison with different optimization techniques. This approach made it possible to evaluate the recommended algorithm consistently and uniformly in comparison with other tested and enhanced methods.

The proposed strategy was compared with four distinct optimization approaches in order to be evaluated including, Emperor Penguin Optimizer (EPO) [26], Spotted Hyena Optimizer (SHO) [27], Multi-Verse Optimizer (MVO) [28], and original Aquila Optimizer (AO) [29]. These algorithms were chosen due to their popularity in the literature and their success in solving optimization issues. The comparison's goal was to assess the effectiveness of the suggested Improved Aquila Optimizer and pinpoint its advantages and disadvantages concerning other cutting-edge algorithms. The value of the variables for the examined methods is shown in Table 1.

**Table 1:** The amount of variables for the examined optimization algorithms.

Algorithm	Parameter	Amount	Algorithm	Parameter	Amount
EPO [26]	$\vec{A}$	[-1.5, 1.5]	MVO [30]	Traveling distance rate	[0.6, 1]
	Temperature value ($T^{\prime }$)	[1, 1000]		Wormhole existence prob.	[0.2, 1]
	M	2			
	f	[2, 3]	SHO [27]	$\vec{M}$	[0.5, 1]
	S	[0, 1.5]			
	l	[1.5, 2]			

The randomization of the process makes it possible for optimization approaches to fail to produce a globally optimum answer occasionally. They are able to locate a suboptimal answer that is quite near to the ideal one, nonetheless, without any difficulty. Each function was consequently evaluated 25 times. It made it possible to analyze important metrics like the average value (Avg) and standard deviation (SD) values more quickly. While the average number shows the mean outcomes for the 25 runs, the standard deviation aids in analyzing the findings' volatility. A mathematical evaluation of the Improved Aquila Optimizer (IAO) with alternative approaches is also included in Table 2.

**Table 2:** A mathematical evaluation of Improved Aquila Optimizer (IAO) with other algorithms.

Test function		IAO	EPO [26]	SHO [27]	MVO [28]	AO [29]
F1	Avg	0.00	3.59	4.75	5.79	3.37
	SD	0.00	3.09	4.51	4.62	2.85
F2	Avg	2.53	3.46	3.92	4.17	3.26
	SD	2.19	3.28	3.61	4.05	3.15
F3	Avg	0.00	1.58e-9	3.67e-7	4.96e-8	4.25e-9
	SD	0.00	0.01	3.65e-6	4.11e-7	5.92e-8
F4	Avg	0.00	0.00	9.52e-5	8.94e-6	2.29e-7
	SD	0.03	0.04	5.83e-4	6.96e-5	10.93e-7
F5	Avg	0.00	3.35	4.45	5.79	3.53
	SD	0.00	2.81	3.92	5.23	3.24
F6	Avg	0.04	6.48	7.11	7.54	5.70
	SD	0.00	6.22	6.82	7.29	5.53
F7	Avg	0.00	5.55	5.64	5.95	5.36
	SD	0.72	5.16	5.25	5.42	4.92
F8	Avg	0.00	0.92	1.83	2.21	0.09
	SD	0.00	0.51	1.32	1.89	0.01
F9	Avg	0.00	0.61	0.96	1.16	0.00
	SD	0.00	0.34	0.67	0.98	0.00
F10	Avg	0.00	0.51	2.93	3.45	1.82
	SD	0.00	0.42	2.72	3.17	1.41

Looking at the results in Tables 2 and it is evident that the IAO algorithm outperforms other algorithms in terms of accuracy when solving the CEC-BC-2017 test suite. The Avg values for IAO are consistently lower than those for the other algorithms, indicating better optimization results. For example, on Test Function F1, IAO achieves an average value of 0.00, while the next best algorithm, EPO, has an average value of 3.59.

Moreover, the IAO algorithm exhibits one of the lowest standard deviation values among the evaluated algorithms. The SD values for IAO are generally smaller than those for the other algorithms, indicating improved reliability and consistency in producing optimal solutions across multiple iterations. The IAO algorithm consistently delivers reliable and trustworthy results throughout various optimization assessments. In summary, the results in Table 2 demonstrate that the Improved Aquila Optimizer (IAO) algorithm performs better than alternative approaches in terms of accuracy and reliability. Its superior performance and lower standard deviation values indicate that the IAO algorithm is capable of consistently producing high-quality results for optimization problems across different test functions.

### 2.4 Objective function and constraints

#### 2.4.1 NET present cost

The NPC stands for a financial element, which is regarded as the research's objective function. The goal of this study is to reduce the total NPC expenses throughout the course of the project. The NPC is determined by [equation (34)]:

(34) $$NPC=C+OM+R+{FC}_{dg}$$

Where, C stands for capital expenses, OM illustrates operations and maintenance, R depicts replacement costs, and ${FC}_{dg}$ depicts fuel costs.

#### 2.4.2 Photovoltaic and the windmill's cost

Costs for Photovoltaic and Windmill are modeled equally. The starting price of the Photovoltaic or Windmill and the area ($A_{PV,WT}$) are used to compute the cost of capital ($C_{PV,WT}$) [equation (35)].

(35) $$C_{PV,WT}=\lambda_{PV,WT}\times A_{PV,WT}$$

The operating and maintenance expenses ($OM_{PV,WT}$) are determined as follows [equation (36)]:

(36) $${OM}_{PV,WT}=\theta_{PV,WT}\times A_{PV,WT}\times \sum_{i=1}^{N}{\left(\frac{1+u}{1+u}\right)}^{i}$$

Where, $\theta_{PV,WT}$ and N are the project's duration and the yearly operating and maintenance expenses for every element, respectively. Since project's duration and the PV or WT duration are equal, the replacement prices are zero.

#### 2.4.3 Diesel cost

The following formulation is a model for the diesel generator's costs [equations (37), (38), (39), (40), (41)]:

(37) $$C_{dg}=\lambda_{dg}\times P_{dg}$$

(38) $${OM}_{dg}=\theta_{dg}\times N_{run}\times \sum_{i=1}^{N}{\left(\frac{1+\mu }{1+i_{r}}\right)}^{i}$$

(39) $$R_{diesel}=R_{dg}\times P_{dg}\times \sum_{i=7,14,..}{\left(\frac{1+\delta }{1+i_{r}}\right)}^{i}$$

(40) $$C_{f\left\{t\right\}}=P_{f}\times F_{dg}\left\{t\right\}$$

(41) $${FC}_{dg}=\sum_{t=1}^{8760}C_{f\left\{t\right\}}\times \sum_{i=1}^{N}{\left(\frac{1+\delta }{1+i_{r}}\right)}^{i}$$

In this context, the variable, $C_{dg}$ refers to the investment cost associated with diesel, $\lambda_{dg}$ shows basic diesel cost, The variable ${OM}_{dg}$ shows operation and replacement cost, $\theta_{dg}$ shows yearly diesel operation and maintenance cost, $N_{run}$ represents the number of times the diesel is run in a year, $R_{diesel}$ shows the cost of replacing diesel, $R_{dg}$ indicates the cost of replacing diesel yearly, and $P_{f}$ shows the cost of fuel. Fuel consumption is calculated using the two parameter $F_{dg}$ and ${FC}_{dg}$.

#### 2.4.4 BESS cost

The costs associated with both the initial installation and ongoing operation and maintenance, including any necessary replacements, of the Battery Energy Storage System (BESS), can be expressed in the following manner [equations (42), (43)]:

(42) $$C_{BESS}=\lambda_{bat}\times C_{bat}$$

(43) $${OM}_{BESS}=\theta_{bat}\times C_{bat\times }\sum_{i=1}^{T_{B}}{\left(\frac{1+\mu }{1+\delta }\right)}^{\left(i-1\right)N_{bat}}$$

Here, $\lambda_{bat}$ represents the primary cost of the Battery Energy Storage System (BESS), while $\theta_{bat}$ represents the annual cost of operating and maintaining the BESS.

#### 2.4.5 The costs associated with the inverter

The costs associated with both the initial investment and ongoing operation and maintenance (O&M) of the inverter are represented in the following manner [equations (44), (45)]:

(44) $$C_{inv}=\lambda_{inv}\times P_{inv}$$

(45) $${OM}_{inv}=\theta_{inv}\times \sum_{i=1}^{N}{\left(\frac{1+\mu }{1+i_{r}}\right)}^{i}$$

The variables in the equation are as follows: $\lambda_{inv}$ represents the initial cost of the inverter, while $\theta_{inv}$ represents the annual cost of Operating And Maintaining (O&M) of the inverter.

### 2.5 The Leveled Cost of Energy index

The Leveled Cost of Energy (LCOE) is a crucial factor representing the cost of producing energy, and is calculated as follows [equation (46)]:

(46) $$LCOE=\frac{NPC\times CRF}{\sum_{i=1}^{8760}P_{load\left\{t\right\}}}$$

Where, CRF refers to the Capital Recovery Factor, which is used to convert the primary cost of a project into an yearly capital cost; $P_{load\left\{t\right\}}$ shows Power load. The CRF is determined as follow [equation (47)]:

(47) $${CRF}_{ir,R}=\frac{i_{r}\times {\left(1+i_{r}\right)}^{R}}{{\left(1+i_{r}\right)}^{R}-1}$$

### 2.6 LPSP index

Reliability is a vital aspect of the Microgrid system, and many indices, such as LOLE, LOEE, and ELF, are used to analyze it. The LPSP, on the other hand, is the most effective and extensively used indicator. The LPSP is a mathematical index used to assess the dependability of a Microgrid system. It is determined by the following formula [equation (48)]:

(48) $$LPSP=\frac{\sum_{t=1}^{8760}P_{load\left\{t\right\}}-P_{pv\left\{t\right\}}-P_{WT\left\{t\right\}}+P_{dg,out\;\left\{t\right\}+}E_{bmin}}{\sum_{t=1}^{8760}P_{load\left\{t\right\}}}$$

### 2.7 Renewable energy index

The Renewable Energy Fraction (RF) is a formula for calculating the percentage of renewable energy in a Microgrid system. The RF is written as follows [equation (49)]:

(49) $$RF=\left(1-\frac{\sum_{t=1}^{8760}P_{dg,out\left\{t\right\}}}{\sum_{t=1}^{8760}P_{re\left\{t\right\}}}\right)\times 100$$

Where, $P_{re}$ shows the total sum of power generated from renewable energy sources.

### 2.8 Index measuring availability

The Availability Factor (A) is considered to be a measure of customer satisfaction and reflects the Microgrid's ability to convert the total energy generated to the load demand. The calculation of this factor is as follows [equation (50), (51)]:

(50) $$A=1-\frac{DMN}{\sum_{t=1}^{8760}P_{load\left\{t\right\}}}$$

(51) $$DMN=P_{bmin\left\{t\right\}}-P_{b\left\{t\right\}}-P_{pv\left\{t\right\}}+P_{WT\left\{t\right\}+}P_{dg,out\left\{t\right\}}-P_{load\left\{t\right\}}\times \mu_{\left\{t\right\}}$$

The variables in the equation are as follows:

Pbmin represents the minimum situation of the battery charge; $P_{b}$ represents the battery power, and u is a constant amount that equals one when the load demand is not met, and when it is 0.

### 2.9 Constraints

Constraints are implemented to adjust the various factors of the Microgrid system and enhance the quality of Microgrid services. In this study, the following constraints are proposed [equation (52)]:

(52) $$0\leq A_{pv}\leq A_{PV}^{\max}\;0\leq A_{WT}\leq A_{WT}^{\max}\;0\leq P_{dg}\leq P_{dg}^{\max}\;LPSP\leq {LSPSP}^{\max}\;{RF}^{\min}\leq RF\;A^{\min}\leq A\;{AD}^{\min}\leq AD$$

## 3 Results and discussion

Creating an HRES is a complex process that necessitates an in-depth knowledge of the system's needs, constraints, and objectives. To obtain good results, it is critical to choose the proper tools and resources. A number of programs, such as Homer and iHoga, have been proposed to improve HRES design; however, because of their structural constraints, they can sometimes fail to supply the specific goals of the project. In order to guarantee that the final system is optimized for efficiency, cost-effectiveness, and sustainable development, it is critical to carefully evaluate the various options and select the proper tool that can effectively handle the unique objectives of the HRES design project. As a result, this research introduces a novel optimization approach that is an enhanced version of Aquila Optimization. A hybrid system of renewable energies has been implemented to demonstrate the efficacy of the suggested algorithm. There are two configurations of the systems under consideration. One consists of a wind turbine, a diesel engine, a battery, and solar energy, while the other is composed of solar energy, a diesel engine, and a battery. This project's objective is to supply the energy demand of households, considering the financial and technological factors of the Golmud. This project can help improve the quality of life of residents and contribute to the general development of Golmud by providing reliable and stable electricity to residential areas.

### 3.1 WT-diesel- battery- PV HRES design

The initial configuration investigated in this study for power supply is a hybrid of PV (Photovoltaic), wind turbine, diesel, and battery technologies. This design has been selected to take advantage of the synergistic effects between PV and WT, which are complementary sources of energy. When these technologies are integrated, their strengths and limitations may be balanced, resulting in a more robust and dependable electrical supply system. Table 3 demonstrates the project, including Net Present Cost (NPC), Renewable Fraction (RF), Power Availability (PA), Loss of Power Supply Probability (LPSP), and Leveled Cost of Energy (LCOE).

**Table 3:** Results of optimization method in first configuration.

Optimization methods	NPC ($)	LOCE ($/Kwh)	LPSP	RF(%)	PA(%)
MVO	284,361	0.2317	0.04286	67.938	98.364
SHO	254,471	0.2301	0.04312	71.452	98.647
EPO	239,582	0.2294	0.04363	79.329	98.851
AO	227,684	0.2279	0.04459	80.174	99.154
IAO	201,973	0.2252	0.04427	82.394	99.214

According to the results, the IAO algorithm is found to be the most suitable for the project with an estimated NPC of 201,973 $, which translates to an LCOE of 0.2252($/kWh). This algorithm also meets all the constraints of the project, including a 0.4427 % LPSP, an 82.394 % Renewable Fraction (RF), and a 99.214% Power Availability (PA). In comparison, the other algorithms that have been used, including MVO, SHO, EPO, and AO, obtained NPC values of 284,361($), 254,471 ($), 239,582 ($), and 227,684 ($), respectively. These results indicate that the IAO algorithm outperformed the other algorithms in terms of economic feasibility and meeting the project's constraints. Choosing the appropriate algorithm for analyzing the feasibility of renewable energy projects is crucial, as it can have a significant impact on the optimization of the system's design and operation, efficient resource utilization, and achievement of desired economic and environmental outcomes. The choice of the IAO algorithm emphasizes the significance of this aspect.

The size of different HRES components is determined by the project's special electricity demand as well as the region's financial and ecological constraints. It is essential to remember that the size of each component is interconnected, and changes in one can have a big influence on the others. As a result, it is critical to choose an algorithm that takes into account all important elements and gives an ideal solution for the entire system. Table 4 illustrates the optimal size of key components of the renewable energy system as determined by the various algorithms employed in the research. The appropriate size of each component is crucial for guaranteeing the system runs smoothly and efficiently while satisfying the community's energy demands.

**Table 4:** Results of the optimal sizing of various components by metaheuristic algorithm.

Optimization methods	PV (m^2^)	WT(m^2)^	Battery(kWh)	Diesel (kW)
MVO	197.658	391.548	24.961	21.487
SHO	172.483	342.154	20.473	22.941
EPO	159.746	252.78	15.457	19.364
AO	112.367	249.17	10.494	15.482
IAO	98.361	154.26	8.127	12.785

According to the findings shown in Tables 2 and it can be said that the IAO method provides the most efficient system sizing. This algorithm proposes a PV area of 98.361 $m^{2}$, a wind area of 154.26 $m^{2}$, a battery with a capacity of 8.127 kWh, and a nominal diesel power of 12.785 kW. The IAO algorithm's optimal size of system components is a critical stage toward the effective deployment of the HRES. The system can run efficiently, meet the energy demands of the residents, and contribute to the general development of the region by choosing the most suitable size values for each component.

The convergence performance is a crucial factor to consider in choosing a suitable optimization algorithm for renewable energy system analysis. A smoother and more stable convergence curve shows that the optimization algorithm is more dependable and can offer an optimized solution for the system more successfully. The success and efficiency of each algorithm are significantly influenced by the convergence rate of each optimization algorithm used in the study of HRES. Fig. (8) shows the convergence curve results for all the algorithms used in the analysis of HRES.

According to Fig. (8), the IAO method provides a superior convergence solution than the other techniques. It is demonstrated through the IAO algorithm's smoother and more stable convergence curve, which suggests a more effective and successful optimization method. The other algorithms, on the other hand, have more unpredictable and unstable convergence curves, showing that they can be less successful at obtaining the best solution for the system. In general, the IAO algorithm's convergence performance lends confidence to its choice as the best method for analyzing the HRES in this study.

Fig. (9 (A-E)) depicts the output electricity of the HRES, including Photovoltaic, Wind, Diesel, and Battery components, throughout a 24-h period, from 900 to 924 h. The goal of this graph is to assess the efficiency of each component of the system and understand how they interact to satisfy the community's demand for electricity.

**Fig. 10:**
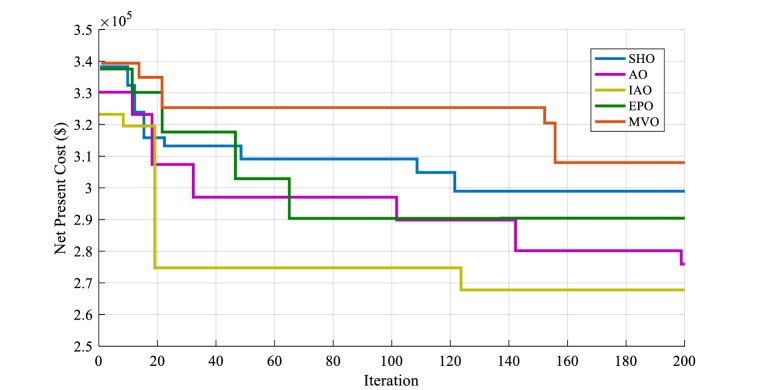
The optimization method convergence rate.

The analysis presented in Fig. (10) provides valuable results about the output of each component of the HRES and how they work together to meet society's energy demand. By understanding the output of each component, it is possible to optimize the design and output of the system, ensure the optimal use of resources, and achieve favorable economic and environmental results. Table 5 presents lifetime costs of the system with the first design.

**Table. (5):** The lifetime costs of the system by the system with the first design.

Optimization algorithm	Lifetime costs				
	PV	WT	Battery	Inverter	Diesel
MVO	900,000	450,000	7000	110,000	230,000
SHO	600,000	300,000	4000	90,000	200,000
EPO	400,000	230,000	3000	70,000	180,000
AO	200,000	70,000	1000	50,000	170,000
IAO	90,000	40,000	0	20,000	165,000

Analyzing the results in Tables 5 and it is evident that the Improved Aquila Optimizer (IAO) algorithm achieves the lowest lifetime costs among all the optimization algorithms considered. The IAO algorithm results in a PV cost of 90,000, a WT cost of 40,000, and zero cost of batteries, indicating that this algorithm generates a more economical system design. Comparatively, the other algorithms demonstrate higher lifetime costs. For example, the most expensive algorithm is the Multi-Verse Optimizer (MVO), which incurs a PV cost of 900,000, WT cost of 450,000, battery cost of 7000, inverter cost of 110,000, and diesel generator cost of 230,000. The second most expensive algorithm is the Shuffled Frog-Leaping Algorithm (SHO) with lifetime costs of 600,000 for PV, 300,000 for WT, 4000 for batteries, 90,000 for inverters, and 200,000 for Diesel Generators. The Evolutionary Programming Optimization (EPO) algorithm yields lower lifetime costs compared to MVO and SHO but is still higher than IAO. The costs for EPO include 400,000 for PV, 230,000 for WT, 3000 for batteries, 70,000 for inverters, and 180,000 for Diesel Generators. Similarly, the Artificial Bee Colony (ABO) algorithm also results in higher lifetime costs, with values of 200,000 for PV, 70,000 for WT, 1000 for batteries, 50,000 for inverters, and 170,000 for Diesel Generators. Therefore, it can be concluded that the cost of diesel is high. The graphical representation shows that the cost of diesel increases significantly compared to other costs related to system performance. However, it is important to include the cost of diesel in the analysis, as it plays an important role in meeting the predetermined system constraints.

### 3.2 Diesel-battery-PV HRES design

In this study, the second design of the HRES uses PV, diesel, and battery technologies, without the use of Wind Turbines. Table 6 demonstrates the numerous algorithms used to assess the financial sustainability of the project for the second configuration of the HRES.

**Table 6:** Results of optimization method in first configuration.

Optimization methods	NPC ($)	LOCE ($/Kwh)	LPSP	RF(%)	PA(%)
MVO	314,482	0.2704	0.5223	53.264	98.139
SHO	300,953	0.2681	0.5364	67.856	98.364
EPO	294,729	0.2594	0.5371	70.357	98.521
AO	273,617	0.2434	0.6460	73.684	99.014
IAO	258,522	0.2452	0.6438	76.329	99.129

The results of the analysis for this configuration show that the most cost-effective investment project is achieved using the IAO algorithm. The project has an estimated cost of 258,522 $, which translates to an LCOE of 0.2452 kWh$. The selection of the IAO method is based on its ability to provide the optimal solution for the HRES and provides the financial and technical considerations of the region. The IAO method is able to recognize the most cost-effective hybrid of solar panel, diesel, and battery technologies while confirming that the system meets all project constraints.

The sizing of each component is an important feature of renewable energy system design since it significantly impacts the system's reliability, efficiency, and cost-effectiveness. Table 7 summarizes the best parameters' size of PV area and diesel capacity for the PV, diesel, and battery HRES system, examined in the present study.

**Table 7:** Best size of parameters PV-battery-diesel.

Optimization methods	PV (m^2^)	Battery(kWh)	Diesel (kW)
MVO	217.821	8.317	22.841
SHO	212.105	7.624	23.634
EPO	209.694	5.571	20.529
AO	192.247	2.694	17.744
IAO	167.637	0.021	15.301

The results suggest that the best configuration for this system requires a PV area of 167.637 m2 and a diesel capacity of 15.301 kW. The IAO algorithm generated these size parameters, which take into account the project's energy demands as well as the region's financial and ecological constraints. Particularly, battery storage is optional for this configuration since diesel generators are designed to supply sufficient additional electricity in the event of a PV system failure. Therefore, the battery storage component, which is often a critical component of renewable energy systems, may only be required in some situations. Instead of incurring the additional costs associated with battery storage, backup diesel generators can be used to ensure successful and efficient system operation.

Fig. (10) shows the convergence performance of the optimization method utilized in the analysis of the second design.

**Fig. 11:**
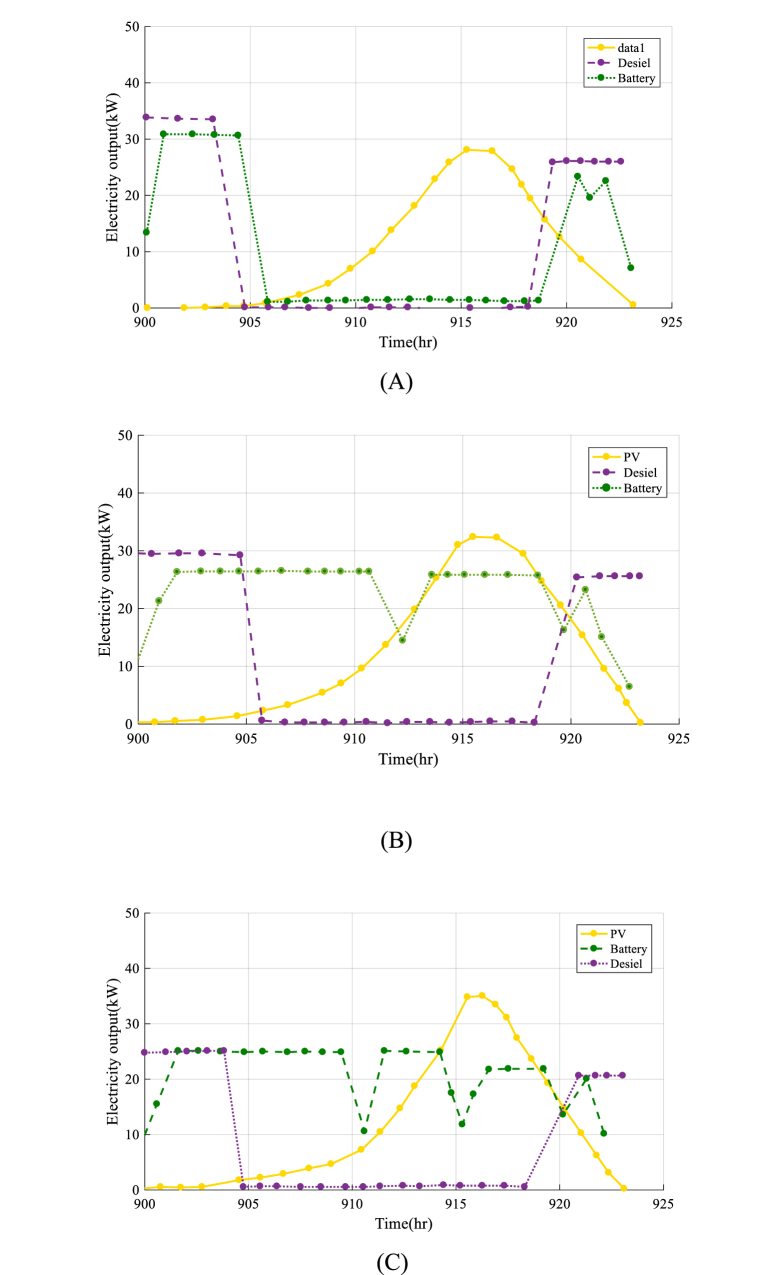

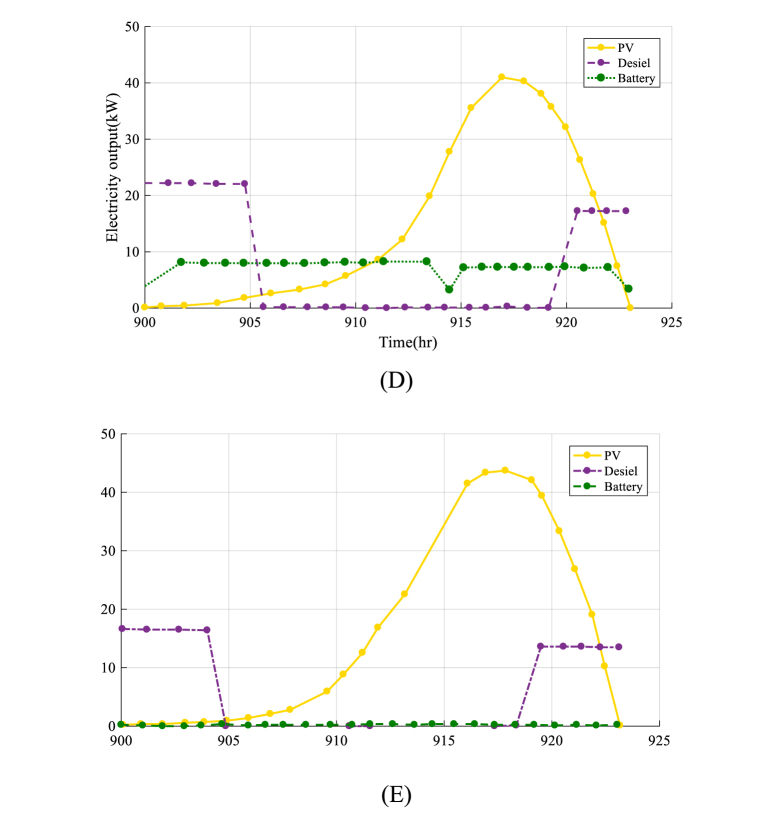
The optimal output electricity of each component in second design of HRES by MVO optimization algorithm(A), SHO optimization algorithm (B), EPO optimization algorithm (C), AO optimization algorithm (D), IAO optimization algorithm (E).

The results presented in Fig. (11 (A-E)) show that the IAOA algorithm performs exceptionally well in finding the best solution for the system. The convergence curve for the IAOA algorithm is smooth and stable, indicating that the algorithm can efficiently optimize the design and operation of the system.

The application of an electricity management system is crucial to ensure that the HRES functions efficiently and successfully. The electricity management system is in charge of controlling the generated electricity from the system's numerous components, such as Photovoltaic cells, diesel power plants, and battery storage to guarantee that the community's energy demands are satisfied. The daily electrical output of the solar panels/diesel/battery HRES system from 900 to 924 h is shown in Fig. (11). The goal of this graph is to evaluate the electrical control system's activity and understand how the system regulates output power during the day.

The findings shown in Fig. (11) show that the electricity management system is operating well to maximize the system's electrical output while accounting for energy consumption, available resources, and environmental circumstances. It is a critical aspect of the economic and environmental viability of the HRES.

Table 8 provides a comprehensive breakdown of the costs incurred over the lifetime of the HRES. This detailed assessment is of great importance to ensure the economic viability of the system and to understand the sustainable financial obligations resulting from the implementation of the system.

**Table 8:** A comprehensive breakdown of the costs incurred over the lifetime of the HRES.

Optimization algorithm	Lifetime costs			
	PV	Battery	Inverter	Diesel
MVO	140,000	4000	30,000	210,000
SHO	11,000	2000	28,000	190,000
EPO	92,000	1000	25,000	175,000
AO	78,000	230	20,000	150,000
IAO	61,000	0	15,000	120,000

Analyzing the results in Tables 8 and it is evident that the Improved Aquila Optimizer (IAO) algorithm results in the lowest lifetime costs among all the optimization algorithms considered for the HRES. The IAO algorithm incurs costs of 61,000 for PV, zero cost for batteries, 15,000 for inverters, and 120,000 for Diesel Generators, indicating a more economically efficient system design. Comparatively, the other algorithms demonstrated higher lifetime costs for the HRES. For example, the Multi-Verse Optimizer (MVO) algorithm incurs costs of 140,000 for PV, 4000 for batteries, 30,000 for inverters, and 210,000 for Diesel Generators, which are higher than the IAO algorithm. Similarly, the Shuffled Frog-Leaping Algorithm (SHO) incurs costs of 11,000 for PV, 2000 for batteries, 28,000 for inverters, and 190,000 for Diesel Generators, higher than the costs incurred by the IAO algorithm. The Evolutionary Programming Optimization (EPO) algorithm yields costs of 92,000 for PV, 1000 for batteries, 25,000 for inverters, and 175,000 for Diesel Generators, while the Artificial Bee Colony (ABO) algorithm demonstrates costs of 78,000 for PV, 230 for batteries, 20,000 for inverters, and 150,000 for Diesel Generators. Consequently, it is evident that the cost of diesel is excessively high; however, it remains a crucial element in achieving optimal performance of the HRES. It emphasizes the significance of investigating other options to decrease the reliance on Diesel Generators in sustainable energy systems.

According to the results of the study, the suggested method for analyzing the HRES has been deemed to be effective because it has the potential to be transmitted among multiple search procedures. This adaptability enables a more efficient and successful optimization procedure, emphasizing the significance of constantly investigating and developing novel optimization strategies for HRES. Advanced optimization approaches can be used to improve the system's design and operation, reduce costs, and ensure that the system satisfies the community's energy needs. Overall, the analytical results show the potential of utilizing advanced optimization approaches to increase the performance and efficiency of HRES.

## 4 Conclusion

Growing demand for renewable energy systems is a reaction to worldwide concerns about global warming and sustainable growth. By combining several renewable energy sources to generate a dependable and sustainable supply of electricity, Hybrid Renewable Energy Systems have emerged as a viable alternative to address the energy demands of distant places. This study emphasized the necessity of optimal design of renewable energy sources to meet the rising energy demands. For this purpose, the Improved Aquila Optimization (IAO) approach is suggested. The implementation of the IAO optimization procedure resulted in a noteworthy decrease in the aggregate expenses of the system, achieving an approximate Net Present Cost (NPC) of $201,973. It represented a cost savings of 25% compared to traditional optimization procedures. Moreover, our study demonstrated a positive impact on the utilization of renewable energy sources, with a notable 15% improvement in the total efficiency of energy production due to the implementation of the IAO strategy. The findings of this study showcased the efficacy of Integrated Approach Optimization (IAO) in effectively addressing the challenges encountered in the design of Hybrid Renewable Energy Systems (HRES). By implementing cost-reduction measures and enhancing operational efficiency, the IAO approach enables the deployment of sustainable energy systems in geographically isolated regions. These findings highlight the importance of employing sophisticated optimization methods like Integrated Assessment and Optimization (IAO) to ensure the economic feasibility and ecological sustainability of Hybrid Renewable Energy Systems (HRES). As a result, the current study emphasized the importance of continuing research and development of novel optimization strategies to address the problems of building HRES and facilitate the transition to clean and sustainable energy systems.

## Data availability statement

Research data are not shared.

## CRediT authorship contribution statement

**Yin Zhou:** Conceptualization, Data curation, Formal analysis. **Zhimin Chen:** Conceptualization, Formal analysis, Resources, Software, Writing – review & editing. **Ziwei Gong:** Conceptualization, Data curation, Methodology, Resources, Software, Writing – review & editing. **Ping Chen:** Conceptualization, Data curation, Formal analysis, Methodology, Software, Supervision, Writing – review & editing. **Saeid Razmjooy:** Conceptualization, Data curation, Formal analysis, Resources, Software, Writing – original draft, Writing – review & editing.

## Declaration of competing interest

The authors declare that they have no known competing financial interests or personal relationships that could have appeared to influence the work reported in this paper.
